# Quantification of Phenolic Compounds and In Vitro Radical Scavenging Abilities with Leaf Extracts from Two Varieties of *Psidium guajava* L.

**DOI:** 10.3390/antiox7030034

**Published:** 2018-02-27

**Authors:** Julio César Camarena-Tello, Héctor Eduardo Martínez-Flores, Ma. Guadalupe Garnica-Romo, José Saúl Padilla-Ramírez, Alfredo Saavedra-Molina, Osvaldo Alvarez-Cortes, María Carmen Bartolomé-Camacho, José Octavio Rodiles-López

**Affiliations:** 1 Programa Institucional de Doctorado en Ciencias Biológicas, Universidad Michoacana de San Nicolás de Hidalgo, Morelia 58240, Mich., Mexico; cama.de.arena@hotmail.com; 2 Facultad de Químico Farmacobiología, Universidad Michoacana de San Nicolás de Hidalgo, Morelia 58240, Mich., México; carbarcam@hotmail.com (M.C.B.-C.); tavarodiles@hotmail.com (J.O.R.-L.); 3 Facultad de Ingeniería Civil, Universidad Michoacana de San Nicolás de Hidalgo, Morelia 58240, Mich., México; ggarnica@umich.mx; 4 Instituto Nacional de Investigaciones Forestales, Agrícolas y Pecuarias, Centro de Investigación Regional Norte-Centro, Campo Experimental Pabellón, Pabellón de Arteaga 20660, Aguascalientes, Mexico; padilla.saul@inifap.gob.mx; 5 Instituto de Investigaciones Químico Biológicas, Universidad Michoacana de San Nicolás de Hidalgo, Morelia 58030, Mich., Mexico; saavedra@umich.mx; 6 Departamento de Bioquímica, Instituto Tecnológico de Morelia, Morelia 58120, Mich., Mexico; oalvarezc@tecmor.mx

**Keywords:** *Psidium guajava*, ORAC, DPPH, ABTS, total polyphenols, total flavonoids, nano spray-dryer

## Abstract

Guava leaf (*Psidium guajava* L.) extracts are used in both traditional medicine and the pharmaceutical industry. The antioxidant compounds in *P. guajava* leaves can have positive effects including anti-inflammatory, anti-hyperglycemic, hepatoprotective, analgesic, anti-cancer effects, as well as protecting against cardiovascular diseases. In the present study, phenolic compounds and in vitro antioxidant capacity were measured in extracts obtained with polar and non-polar solvents from leaves of two varieties of guava, Calvillo Siglo XXI and Hidrozac. The quantity of total phenolics and total flavonoids were expressed as equivalents of gallic acid and quercetin, respectively. Hydroxyl radical, **2,2′-azino-bis(3-ethylbenzothiazoline-6-sulphonic acid)** (ABTS), 2,2-diphenyl-1-picrylhydrazyl (DPPH), and Oxygen Radical Absorbance Capacity using fluorescein (ORAC-FL) in vitro tests were used to assess the radical scavenging abilities of the extracts. The total phenolics were higher in the aqueous fraction of the variety Calvillo Siglo XXI, while in the Hidrozac variety total phenolics were higher in the acetone and chloroform fractions. Total flavonoids were higher in all fractions in the variety Calvillo Siglo XXI. Total phenolics showed a highly positive correlation for ORAC-FL, and a moderately positive correlation with hydroxyl radicals. Finally, total flavonoids showed a slightly positive correlation for ORAC-FL and hydroxyl radicals. Both varieties of guava leaf extract showed excellent antioxidant properties.

## 1. Introduction

*Psidium guajava* L. is a small tree that belongs to the Myrtaceae family. Guava is the common name used for species of *P. guajava*. The guava tree is distributed in tropical and subtropical regions of America, the Caribbean, Asia, Africa, and the Pacific islands [1]. The primary producers of guava fruit are India, China, Thailand, Pakistan, and Mexico [2,3].

Currently, most of the commercialized orchards of guava fruits in Mexico present a great phenotypic, genetic, and morphological diversity due to propagation methods. This variability affects the productive potential of the guava crop, as well as the uniformity and quality of the harvested fruit. To improve the productivity of the guava crop in Mexico, the germplasms of different varieties has been selected and evaluated, which will be registered and commercially released as clonal varieties [4,5,6,7].

Different parts of guava trees are widely used as food and in folk medicine around the world [8]. Díaz-De-Cerio [9] reported that several studies have shown that guava leaves present anti-hyperglycemic and anti-hyperlipidemic activities. In Mexico, a phytodrug QG-5^®^ is being produced from leaves of *Psidium guajava folia,* variety [10]. The activities present in extracts of guava leaves are mainly due to phenolic compounds.

Water and organic solvents such as chloroform, hexane, ethyl acetate, acetone, methanol, and ethanol are the most commonly used solvents for extracting phenolic compounds from leaves of different species of trees or plants. The nature of the sample as well as the polarity of the solvents are two of the main factors that influence the solubility of phenolic compounds [11,12,13,14].

Seo et al. [15] described that water, followed by ethanol and finally methanol, are the best solvents used to obtain extracts with high antioxidant capacity, in leaves of *Psidium guajava*. A study conducted by Nantitanon et al. [16] found that the extraction of phenolic compounds with greater antioxidant activity were obtained using the ultrasound process, followed by maceration with agitation, maceration without agitation, and finally soxhlet extraction. In other studies, the antioxidant properties related to phenolic compounds also were documented in extracts of branches, leaves, fruits, and seeds of guava trees [17,18].

According to reports, in the aforementioned studies, different solvents can be used for the extraction of phenolic compounds from guava leaves, but there is a possibility that these extracts present a risk to consumers. With respect to the toxicity of guava leaf extracts, Chen et al. [19] documented that ethanol and acetone extracts at concentrations greater than 100 μg/mL and 50 μg/mL, respectively, may present cytotoxicity, while aqueous extracts at concentrations lower than 500 μg/mL do not represent cytotoxicity.

The main phenolic compounds reported in guava leaves extracts are gallic acid, catechin, chlorogenic acid, caffeic acid, epicatechin, rutin, quercetin, kaempferol, and luteolin [20]. The phenolic compounds in guava leave extracts may vary depending on the variety, the way to dry the leaves, extraction technique, and the maturity of the leaves [16,21,22].

Consumption of antioxidants significantly decreases the adverse effects of some reactive oxygen and nitrogen species in the human body. The antioxidant capacity of the extracts can be measured by in vitro assays, which can be classified as either assays based on hydrogen atom transfer (HAT) or assays based on electron transfer (ET). The oxygen radical absorbance capacity (ORAC) is classified as HAT type assay, while 2,2-diphenyl-1-picrylhydrazyl (DPPH), total phenols, **2,2′-azino-bis(3-ethylbenzothiazoline-6-sulphonic acid)** (ABTS), and hydroxyl radicals are classified as electron transfer (ET)-type assays [23,24].

Total phenols, DPPH, hydroxyl radicals, and ABTS are the main assays that are carried out with the extracts of leaves form different varieties of *Psidium guajava*. Siwarungson et al. [22] used three varieties of *Psidium guajava* grown in Thailand, measuring total phenols, DPPH, and hydroxyl radicals. Jeong et al. [21] measured total phenolic compounds, ABTS, and DPPH in three varieties of *Psidium guajava* cultivated in Korea. These assays are used due to the versatility of the samples that can be analyzed from cell lines to nutrition lines, since it shows a premise of the structure and its relationship in the antioxidant activity of the compounds contained in the samples [25,26].

The objective of the present work was to quantify total polyphenols, total flavonoids, and the in vitro radical scavenging abilities of extracts obtained from Calvillo Siglo XXI and Hidrozac variety guava leaves. Currently, in Mexico these varieties do not have information regarding their content of total polyphenols, total flavonoids, and their antioxidant activity of their leaves. The only study that counts in Mexico of the variety Hidrozac and Calvillo is with its antioxidant activity of its fruit [27].

These varieties were registered in Mexico by the INIFAP (National Institute of Forestry, Agricultural and Livestock Research, Pabellón de Arteaga, Aguascalientes, México) in 2009 [4]. A difference between these two varieties is that Hidrozac produces pink pulp fruit while Calvillo Siglo XXI produces yellow pulp fruit. In the present work, it was found that there were significant differences in the amount of phenolic compounds and the radical scavenging abilities of Calvillo Siglo XXI and Hidrozac varieties. The correlation between total polyphenols and the radical scavenging abilities of the hydroxyl radicals and the ORAC test was positive.

## 2. Materials and Methods 

### 2.1. Materials

#### Samples

The guava leaves were collected at the experimental site “Los cañones” of the INIFAP (National Institute of Forestry, Agricultural and Livestock Research), which is located in the municipality of Huanusco, Zacatecas, Mexico. Samples were collected 4 September 2015 and 24 August 2016. The coordinates of the collection site are longitude 21° 44.7′ N and 102° 58.0′ W to 1508 masl. Two varieties were selected, which are registered in the catalogue of vegetal varieties from 2009. Calvillo Siglo XXI no. GUA-005-160709 which produces yellow pulp fruits and Hidrozac no. GUA-002-160709 which produces pink pulp fruit. The collected leaves were dried in the shade and milled with a knife mill (PULVEX model 200, PULVEX SA. CV., Mexico City, Mexico) using a 0.8 mm diameter mesh. The ground material was stored in plastic bags in the dark at 22 °C prior to extraction.

### 2.2. Methods

#### 2.2.1. Extractions

In all cases, regardless of the solvent used, the guava leaves were macerated under discontinuous stirring, with double extraction period (24 h × 2) at a solvent: sample ratio 10:1 (*v*/*w*). The samples collected in 2015 were subjected to extractions with solvents of different polarities: chloroform, acetone and water. For the extracts of the leaves collected in 2016, the samples were macerated only with water. The use of chloroform and acetone, and water, was to know the performance of the extracts before different solvents, and to know their composition in terms of total phenolic compounds and total flavonoids, as well as their antioxidant capacity. However, for the study of the leaves collected in 2016, it was decided to work only with water as a solvent, since it is the extract that would not present a possible toxicological effect, having the prospect of testing it on living organisms. In this way, we can have comparisons between the extracts obtained from the leaf collected both in 2015 and in 2016 using water as a solvent. Chloroform and acetone were recovered in a rotary evaporator (BM200, Yamato Scientific America Inc., Santa Clara, CA, USA) at 35 °C in a water bath, and the residual material was dried at 40 °C. The aqueous extract was dried using a nano spray dryer (B-90, BÜCHI Labortechnik AG, Flawil, Switzerland) using the following conditions: inlet temperature 105 °C, outlet temperature 38 °C, 90% sprinkler, and a 7 µm spray cap. Briefly, the liquid sample was fed to the spray head and droplets were generated by a piezoelectric driven actuator in a stainless steel membrane with holes of 7.0 μm inside the spray cap. The droplets were dried and the particles collected by electrostatic charging to deflect them to the collecting electrode. Nanometric particles were then collected. The dried extracts obtained for each solvent were stored in glass jars in the dark until use.

#### 2.2.2. Total Phenolics

The total phenolic compounds were quantified using the methodology of Singleton et al. [28] and Taga et al. [29] with some modifications. 100 µg/mL of extracts was dissolved in 80% MeOH. Then, 100 µL of the solution was added to 100 µL of Folin–Ciocalteu reagent (1 N). This mix was stirred. After 3 min at rest, 2 mL of 2% *m*/*v* Na_2_CO_3_ was added and stirred and incubated in the dark for 30 min. Absorbance was then measured at 750 nm in a spectrophotometer UV–vis (LAMBDA 365, PerkinElmer, Inc., Waltham, MA, USA). The results were expressed as milligrams equivalent of gallic acid per gram of extract (mg GAE/g). Determinations were carried out in triplicate. A calibration curve was constructed using gallic acid at 0–4 µg.

#### 2.2.3. Total Flavonoids

Quantification of flavonoids was carried out using the methodology of Dewanto et al. [30] with some modifications. 100 µg per mL of extracts were dissolved in 80% MeOH. After, 250 µL of the solution were added to 1.250 µL of H_2_O, and 75 µL of NaNO_2_ at 5% m/v. After 6 min 150 µL of 10% m/v AlCl_3_ was added. After another 5 min 500 µL of 1 M NaOH and 275 µL of H_2_O were added. Subsequently, absorbance was measured at 510 nm in a UV–vis spectrophotometer (LAMBDA 365, PerkinElmer, Inc., Waltham, MA, USA). Results were expressed as milligrams equivalent of quercetin per gram of extract (mg QE/g) and determinations were made in triplicate. A calibration curve was constructed using quercetin at 0–30 µg.

#### 2.2.4. Hydroxyl Radicals

The ability to scavenge hydroxyl radicals was determined using the methodology of Smirnoff and Cumbes [31] and Zhang et al. [32], with some modifications. 8 mM FeSO_4_, 3 Mm C_7_H_6_O_3_, and 2% H_2_O_2_ were prepared with distilled water. The extracts were dissolved in **dimethyl sulfoxide** (DMSO) at a concentration of 500–3000 µg/mL. The reaction mixture consisted of 667 µL of extract, 200 µL of FeSO_4_, 167 µL of H_2_O_2_, and 667 µL of C_7_H_6_O_3_. The mixture was placed in a water bath for 30 min at 37 °C, before 299 µL of distilled water was added and the absorbance was measured (A1) at a wavelength of 510 nm on a UV–vis spectrophotometer (LAMBDA 365, PerkinElmer, Inc., Waltham, MA, USA). The ability to scavenge hydroxyl radicals was calculated using the following formula (1) used by Fu et al. [33]

(1) $$\mathrm{HO}\;\%=\frac{A0-\left(A1-A2\right)}{A0}\times 100\%$$

where A0 is the absorbance of the control group (the sample solution was replaced with dilution solvent) and A2 the absorbance of the blank (H_2_O and sample solution). The half maximal inhibitory concentration (IC_50_) was calculated.

#### 2.2.5. ABTS

The ABTS was measured according to the methodology of Re et al. [34] and Tachakittirungrod et al. [35] with some modifications. A calibration curve was generated with Trolox and results were expressed as TEAC (trolox equivalent antioxidant capacity). ABTS was dissolved in distilled water at a concentration of 7 mM and mixed with a 2.45 mM solution of potassium persulfate in water. The mixture was left to stand for 15 h in the dark to generate the radical solution (ABTS•^+^). For the tests, a fraction of the radical solution (ABTS•^+^) was prepared by adding MeOH until an absorbance of 0.7 ± 0.2 at a wavelength of 750 nm was reached. 180 µL of the radical solution (ABTS•^+^) was then mixed with 20 µL of extracts. After 5 min, absorbance was measured at a wavelength of 750 nm in a UV–vis spectrophotometer (LAMBDA 365, PerkinElmer, Inc., Waltham, MA, USA). The extracts were dissolved in MeOH at a concentration of 1 mg/mL.

#### 2.2.6. DPPH

The assay was based on the method described by Brand-Williams [36], with some modifications. The reagents used as well as all the extracts were diluted with MeOH. The concentration of DPPH was $6\times 10^{-5}$ M and ascorbic acid was prepared at concentrations of 50–200 µg/mL. The extracts were prepared at concentrations of 50–1000 µg/mL. For the test, 100 µL of extract or ascorbic acid were mixed with 1900 µL of DPPH solution and held in the dark for 15 min. Absorbance was measured at a wavelength of 515 nm in a UV–vis spectrophotometer (LAMBDA 365, PerkinElmer, Inc., Waltham, MA, USA). Ascorbic acid was used as a control and was quantified at the half maximal inhibitory concentration (IC_50_).

#### 2.2.7. Oxygen Radical Absorbance Capacity Using Fluorescein (ORAC-FL)

The ORAC-FL test was conducted using the method described by Dávalos et al. [37] and Ou et al. [38] with some modifications. A 75 mM phosphate buffer solution (PH 7.4) was prepared. This buffer was used to prepare 70 nM fluorescein, 12 nM 2,2′-Azobis(2-amidinopropane) dihydrochloride (AAPH), and 2–8 µΜ Trolox. The extracts were diluted with 80% MeOH at a concentration of 10 µg/mL. The test was performed in a 96 well microplate. 120 µL of fluorescein was mixed with 20 µL of sample or trolox and incubated for 30 min at 37 °C. Subsequently, 60 µL of AAPH was added immediately. The fluorescence was measured every minute for 120 min, stirring automatically before each reading. A blank was prepared by replacing the antioxidant with phosphate buffer solution.

The area under the curve of fluorescence was calculated with Equation (2),

(2) $$\mathrm{AUC}=1+\sum_{i=1}^{i=120}f_{i}/f_{0}$$

where $f_{0}$ is the initial fluorescence read at time 0 and $f_{i}$ is the fluorescence read at the time i. The net area under the curve (AUC) of samples was calculated by subtracting the AUC from the blank. Regression equations between net AUC and antioxidant concentration were calculated for all samples. A calibration curve was prepared with Trolox. The results were expressed as micromolar equivalents of Trolox for each microgram of extract (µΜ TE/µg). The measurements of fluorescence were performed at a temperature of 37 °C with an excitation wave of 485 nm and an emission wave of 520 nm in a spectral scanning multimode reader (Thermo Fisher Scientific, Waltham, MA, USA).

#### 2.2.8. Statistical Analysis

All experiments were done in triplicate. The values were presented as the average ± SD (*n* = 3). Significant differences in the groups were determined by the analysis of variance using the Tukey Test HSD (honest significant difference) by homogeneous groups, calculated with Statistica software (version 7 Statistica Ink., Palo Alto, CA 94304, USA). *p* < 0.05 was considered statistically significant. Excel 2016 software (Microsoft Office 365, One Microsoft Way, Redmond, WA, USA) and JMP8 software (SAS, Cary, NC, USA) were used for correlation analysis.

## 3. Results

### 3.1. Total Phenolic and Total Flavonoid Compounds 

Total polyphenols and total flavonoids were quantified in the aqueous, acetone, and chloroform extracts of both varieties of guava leaves.

#### 3.1.1. Total Phenolic Compounds

Table 1 shows results of total phenolic compounds. Acetone had the greatest quantity of phenolic compounds for both varieties, followed by the aqueous fraction, and finally the chloroform fraction. Between the two varieties, the chloroform extracts were statistically equivalent. In the acetone fraction, the extracts of Hidrozac had more phenolic compounds (374.63 mg GAE/g extract), whereas in the aqueous fraction Calvillo Siglo XXI had a greater quantity (242.10 mg GAE/g extract). The total phenolic compounds in the extract of the acetone fraction for both varieties was more than double the 141 mg EAG/g measured by You et al. [18] who also extracted with acetone for one night following maceration. The phenolic compounds obtained in our aqueous extracts were greater than those quantified by Seo et al. [15] from extracts boiled for 4 h in guava leaves (140 mg gallic acid equivalent (GAE)/g).

#### 3.1.2. Total Flavonoids

Table 2 shows total extracted flavonoids. The extract with the greatest quantity of flavonoids, in general, was the acetone fraction, followed by the aqueous fraction, and finally the chloroform fraction. Between the two varieties, total flavonoid content in the chloroform fraction was statistically equivalent, while in the aqueous fraction and in the acetone fraction, Calvillo Siglo XXI had a greater quantity of total flavonoids. Total flavonoids in the aqueous fraction measured in our study was greater than the 50 mg QE value for aqueous extraction reported by Seo et al. [15]. Rattanachaikunsopon and Phumkhachorn [39] measured flavonoid contents of fresh and dry guava leaves by drying leaves for 72 h at 70 °C, extracting with methanol, and purifying using a chromatographic column. They found that flavonoid content was higher in dry leaves and that quercetin was the most abundant flavonoid.

### 3.2. Radical Scavenging Ability In Vitro

The assays for radical scavenging ability in vitro were conducted with aqueous, acetone, and chloroform leaf extracts of both guava varieties.

#### 3.2.1. Hydroxyl Radicals

In general, the chloroform fraction had the lowest IC_50_, followed by the aqueous fraction, and finally the acetone fraction (Table 3). Between the two varieties, chloroform or acetone showed no significant differences (*p* < 0.05), while the aqueous fraction did show significant differences (*p* < 0.05). The variety Calvillo Siglo XXI had a lower IC_50_ in both the aqueous and chloroform fractions, while the Hidrozac variety had the lowest IC_50_ in the acetone fraction. The IC_50_ values measured in the present work are up to 20 times higher than those values reported by Kim et al. [40] for aqueous extracts obtained by maceration for 24 h (110 µg/mL). A half maximal effective concentration (EC_50_) of 5470–6700 for 50 µg/mL extracts was reported by Ademiluyi et al. [41], who used a mixture of MeOH and HCl for extraction of four different varieties of macerated guava.

#### 3.2.2. ABTS

The results of ABTS radical scavenging tests are described in Table 4. The results were expressed as the antioxidant capacity equivalent to a millimolar of trolox per milligram of extract (TEAC mM/mg). In general, the extracts with the greatest TEAC were obtained from acetone fractions, followed by the chloroform fractions, and finally the aqueous fractions. Between the two varieties, only the aqueous fraction showed significant differences (*p* < 0.05). In the study carried out by Díaz-De-Cerio et al. [42] with aqueous extracts of guava leaves obtained by sonication for 10 min and infusion at 3.5 and 7 min, TEAC values ranged from 0.18 to 1.13. The values from that study are two to four times lower than the aqueous fraction values measured in the present work.

#### 3.2.3. Radical DPPH

Table 5 shows the results of DPPH radical scavenging ability. The extracts with lower IC_50_ values were those obtained using acetone as a solvent. All the extracts in both varieties were significantly different (*p* < 0.05). The IC_50_ of the extracts of the acetone fraction obtained in this work was almost three times higher than IC_50_ values measured by You et al. [18] (34 µg/mL). The IC_50_ value of the aqueous fraction in the present work were also more than six times higher than the IC_50_ value measured from the aqueous fraction of guava leaves macerated for 24 h by Kim et al. [40] (120 µg/mL). IC_50_ values ranging from 730 to 910 µg/mL for extracts of four varieties of guava leaves macerated for 24 h using a mixture of MeOH/HCl have also been reported by Ademiluyi et al. [41], which are within the range measured in the present work.

#### 3.2.4. ORAC-FL

The results of peroxyl scavenging ability were expressed as micromoles of trolox equivalents per microgram of extract (µm TE/µg) and are shown in Table 6. The extract with the greatest TE was the 2015 aqueous fraction of Calvillo Siglo XXI, with a value of 7.5 µΜ TE/µg. In general, the Calvillo Siglo XXI variety had larger values in water and acetone fractions than Hidrozac. In the chloroform fraction, no significant differences were observed (*p* < 0.05) for both varieties. It is worth mentioning that this is the first study reporting the use of ORAC to measure antioxidant capacity in extracts of guava leaves, obtaining interesting and complementary results to the measurement of antioxidant activity. This test can be recommended to study antioxidant capacity for both guava leaves and other extracts from different plant sources.

### 3.3. Correlation Analysis between Phenolic and Flavonoids Compounds and Radical Scavenging Ability

We observed a correlation between total phenolic compounds, total flavonoid compounds, ABTS, DPPH, radical hydroxyl, and ORAC test (Table 7). Comparing the correlation between the total flavonoids and the rest of the assays, the largest positive correlation was found with total phenolic compounds (0.61), followed by the hydroxyl radical test (0.38), and the ORAC value (0.31). The total flavonoids showed a strong negative correlation with the DPPH assay (−0.76). When total phenolic compounds were correlated with the rest of the assays, the greatest positive correlation was found against the ORAC assay (0.71), followed by the hydroxyl radical assay (0.69). Total phenolic compounds presented a strongly negative correlation with the DPPH assay (−0.59) and a weakly negative correlation with the ABTS assay (−0.15). Total phenolic compounds, ORAC value, and the hydroxyl radical (OH) assay had the highest positive correlations. This indicates that these assays are the most useful to measure antioxidant capacity in vitro from different extracts from leaves of *P. guajava* L. Chen et al. [43] reports a positive correlation between total polyphenols and other assays such as ABTS using guava leaf extracts. The negative correlation between the DPPH assay and total phenolics and total flavonoids found in this work also was reported by Ademiluyi et al. [41]. In addition, they reported a positive correlation between total phenolics and the ABTS assay, contrary to the data obtained in our research.

## 4. Conclusions

Significant differences in guava leaf extracts phenolic compound content and radical scavenging abilities were found between the varieties Calvillo Siglo XXI and Hidrozac. The phenolic and flavonoids contents as well as the ability to scavenging radicals were similar in both varieties, regardless of collection year. Extracts from guava leaves can donate electrons and hydrogen atoms, based on the results of the assays. Total polyphenol, hydroxyl radical, and ORAC assays are recommended for comparing differences between varieties. The novelty of this work is that there are no reports of tests with extracts of guava leaves from the varieties Hidrozac and Calvillo Siglo XXI. In addition, no work has been reported using the ORAC-FL trial with any variety of guava leaves. The results of this study will be complemented by a quantitative analysis of the phenolic compounds present in the different extracts by chromatographic methods. In addition, an in vivo test will be performed with rats in which aqueous extracts will be administered to evaluate their possible antihypertensive effect.

## Figures and Tables

**Table 1:** Total polyphenols in the aqueous, acetone, and chloroform extracts of Calvillo Siglo XXI and Hidrozac leaves. The average values (±SD) are expressed as milligrams equivalent of gallic acid per gram of extract (mg GAE/g).

Extracts		Phenolic Compounds mg GAE/g Extract
Water 2016	Calvillo Siglo XXI	285.21 ± 35.54 ^c,d^
	Hidrozac	256.76 ± 9.62 ^c^
Water 2015	Calvillo Siglo XXI	242.10 ± 13.33 ^c^
	Hidrozac	187.01 ± 9.24 ^b^
Acetone 2015	Calvillo Siglo XXI	314.03 ± 8.05 ^d^
	Hidrozac	374.63 ± 29.92 ^e^
Chloroform 2015	Calvillo Siglo XXI	71.69 ± 3.69 ^a^
	Hidrozac	100.03 ± 2.37 ^a^

Different letters indicate significant differences (*p* < 0.05).

**Table 2:** Total flavonoids in the aqueous, acetone and chloroform leaf extracts of the varieties Calvillo Siglo XXI and Hidrozac. The average values (±SD) are expressed as milligrams equivalent to quercetin per gram of extract (Mg QE/g).

Extracts	Total Flavonoids mg QE/g Extract
Water 2016 Calvillo Siglo XXI	180.77 ± 7.23 ^d^
Hidrozac	152.61 ± 8.14 ^c^
Water 2015 Calvillo Siglo XXI	108.54 ± 3.54 ^b^
Hidrozac	77.83 ± 6.65 ^a^
Acetone 2015 Calvillo Siglo XXI	239.45 ± 11.32 ^e^
Hidrozac	135.88 ± 7.16 ^c^
Chloroform 2015 Calvillo Siglo XXI	107.48 ± 13.30 ^b^
Hidrozac	96.39 ± 4.57 ^a,b^

Different letters indicate significant differences (*p* < 0.05).

**Table 3:** Hydroxyl radical scavenging ability in aqueous, acetone, and chloroform extracts of the leaves of Calvillo Siglo XXI and Calvillo. The average values (±SD) are expressed as half maximal inhibitory concentrations in micrograms per milliliter (IC_50_ µg/mL).

Extracts	Hydroxyl Radical Scavenging IC_50_ µg mL
Water 2016 Calvillo Siglo XXI	1607.81 ± 110.78 ^b,c^
Hidrozac	2360.56 ± 104.80 ^d^
Water 2015 Calvillo Siglo XXI	1726.51 ± 90.58 ^c^
Hidrozac	2355.73 ± 60.07 ^d^
Acetone 2015 Calvillo Siglo XXI	2416.04 ± 114.66 ^d^
Hidrozac	2173.37 ± 108.42 ^d^
Chloroform 2015 Calvillo Siglo XXI	1103.42 ± 28.62 ^a^
Hidrozac	1355.08 ± 134.28 ^b^

Different letters indicate significant differences (*p* < 0.05).

**Table 4:** ABTS radical scavenging ability in the aqueous, acetone, and chloroform extracts of the leaves of Calvillo Siglo XXI and Hidrozac. The average values (±SD) are expressed as antioxidant capacity equivalent to a millimolar of trolox per milligram of extract (TEAC mM/mg).

Extracts	ABTS Radical Scavenging TEAC mM/mg
Water 2016 Calvillo Siglo XXI	2.64 ± 0.09 ^b^
Hidrozac	3.34 ± 0.02 ^c^
Water 2015 Calvillo Siglo XXI	2.37 ± 0.15 ^a^
Hidrozac	4.10 ± 0.03 ^d^
Acetone 2015 Calvillo Siglo XXI	5.28 ± 0.01 ^f^
Hidrozac	5.24 ± 0.04 ^f^
Chloroform 2015 Calvillo Siglo XXI	4.99 ± 0.02 ^e^
Hidrozac	5.13 ± 0.02 ^e,f^

Different letters indicate significant differences (*p* < 0.05); TEAC: trolox equivalent antioxidant capacity). ABTS: **2,2′-azino-bis(3-ethylbenzothiazoline-6-sulphonic acid).**

**Table 5:** DPPH radical scavenging ability in the aqueous, acetone, and chloroform extracts of the leaves of Calvillo Siglo XXI and Hidrozac. The average values (±SD) are expressed as half maximal inhibitory concentration in micrograms per milliliter (IC_50_ µg/mL).

Extracts	DPP Radical Scavenging IC_50_ µg mL
Water 2016 Calvillo Siglo XXI	269.78 ± 12.89 ^d^
Hidrozac	421.77 ± 13.17 ^f^
Water 2015 Calvillo Siglo XXI	671.44 ± 16.36 ^h^
Hidrozac	811.25 ± 7.74 ^i^
Acetone 2015 Calvillo Siglo XXI	141.64 ± 1.66 ^c^
Hidrozac	105.03 ± 1.70 ^b^
Chloroform 2015 Calvillo Siglo XXI	385.15 ± 10.11 ^e^
Hidrozac	608.32 ± 11.81 ^g^
Ascorbic acid	78.60 ± 2.21 ^a^

Different letters indicate significant differences (*p* < 0.05). DPP: 2,2-diphenyl-1-picrylhydrazyl radical.

**Table 6:** Peroxyl radical scavenging ability in aqueous, acetone, and chloroform extracts of the leaves of Calvillo Siglo XXI and Hidrozac. The average values (±SD) are expressed as micromoles trolox equivalents per microgram of extract (µΜ TE/µg).

Extracts	Peroxyl Radical Scavenging µg TE/µg
Water 2016 Calvillo Siglo XXI	4.9 ± 0.75 ^b,c^
Hidrozac	4.6 ± 0.80 ^b,c^
Water 2015 Calvillo Siglo XXI	7.5 ± 0.73 ^d^
Hidrozac	4.5 ± 0.87 ^b^
Acetone 2015 Calvillo Siglo XXI	5.5 ± 0.65 ^c^
Hidrozac	5.0 ± 0.25 ^b,c^
Chloroform 2015 Calvillo Siglo XXI	2.1 ± 0.19 ^a^
Hidrozac	2.1 ± 0.09 ^a^

Different letters indicate significant differences (*p* < 0.05).

**Table 7:** Correlations between phenolic and flavonoid compounds and antioxidant properties of guava extracts.

	T.F.	T.P.	ABTS	DPPH	ORAC	OH
**T.F.**	1					
**T.P.**	0.61	1				
**ABTS**	0.06	−0.15	1			
**DPPH**	−0.76	−0.59	−0.35	1		
**ORAC**	0.31	0.71	−0.58	−0.04	1	
**OH**	0.38	0.69	0.01	−0.14	0.52	1

T.F. = Total flavonoids. T.P. = Total phenolics. OH = Hydroxyl radical. DPPH: 2,2-diphenyl-1-picrylhydrazyl; ORAC: Oxygen Radical Absorbance Capacity.
